# Effect of Wheat Straw as a Cover Crop on the Chlorophyll, Seed, and Oilseed Yield of *Trigonella foeunm graecum* L under Water Deficiency and Weed Competition

**DOI:** 10.3390/plants8110503

**Published:** 2019-11-14

**Authors:** Nabil Raheem Lahmod, Jawadayn Talib Alkooranee, Ahmed Abed Gatea Alshammary, Jesús Rodrigo-Comino

**Affiliations:** 1 Field crops Department, College of Agriculture, University of Wasit, Wasit 00964, Iraq; ja_alja@yahoo.com; 2 Soil Science and Water Resources Departments, College of Agriculture, University of Wasit, Wasit 00964, Iraq; agatea@deakin.edu.au; 3 School of Engineering, Deakin University, Geelong, VIC 3216, Australia; 4 Physical Geography, Trier University, 54286 Trier, Germany; rodrigo-comino@uma.es; 5 Soil Erosion and Degradation Research Group, Department of Geography, University of Valencia, 46010 Valencia, Spain

**Keywords:** mulching system, water resources, *Trigonella foeunm graecum* L, agricultural-management system

## Abstract

The effects of water stress on fenugreek crops are well documented. However, little is known about how these plants respond to water deficits under a soil-mulching system when the surface is protected. Therefore, the current research aims to demonstrate the possibility of reducing the impact of water stress and weed competition on the fenugreek crop through the use of wheat residues as a cover crop on the soil surface. A field experiment was carried out during the winter season (2016–2017) using a split-plot design arrangement with three replicates. The experiments included four levels of water deficit, which consisted of a 40% depletion treatment as a control plot, and 50%, 60%, and 70% depletion from the field capacity (DFC) for the other studied fields. The subplot division consisted of mulching the soil with wheat residues. The results demonstrated that soil-mulching systems and a water deficit are able to impact the fenugreek yield of seed and oil. Additionally, soil mulching led to a decrease in weed density and biomass, chlorophyll content, and biological yield. Although high water deficit (70% DFC) led to yield and growth reduction, the use of wheat residue as a cover crop moderated the effect of a strong water deficit on plants and showed clear reduction of weed growth. Therefore, the results suggest that soil mulching can mitigate the adverse effects of water deficit by conserving soil moisture and decreasing weeds, which can be considered an acclimation mechanism under water-deficit conditions to avoid yield loss. Moreover, the allelopathic effects of wheat residue were observed on fenugreek crops subjected to irrigation after depleting 40% soil water moisture, but these effects disappeared within 90 days of sowing. We conclude that these results can help future agricultural planning and systems in order to increase productivity, reduce irrigation costs, and conserve soil quality.

## 1. Introduction

Fenugreek (*Trigonella foeunm graecum* L) is a legume originally from Western Europe, Mediterranean countries, and Northwest Asia [1]. The traditional importance of fenugreek in these areas is due to its chemical and nutritional contents; proteins comprise 25–30% [2], fats comprise 5.5–7.5% [3], and the proportion of carbohydrate ranges from 45% to 60%. Fenugreek also contains vitamins [4], and many effective medical and pharmaceutical compounds, including trigonelline and choline [5]. This plant has been used for decades to treat diabetes and blood cholesterol [6], for its antioxidant and antibacterial properties [7], and for its potential inhibition of malignant tumors [8]. Fenugreek is one of the most important globally distributed medicinal plants, growing in such locations as Iraq, Lebanon, Egypt, France, and India. Although arid and semiarid environmental conditions are suitable for the growth of this medicinal crop, many problems surrounding its productivity have been detected, such as lack of water, a high degree of soil salinity, growth of weeds, and insects and other pests. 

In particular, water stress is one of the most important abiotic environmental issues because it adversely affects the fenugreek yield, quality, and, subsequently, its medical properties. Some authors [9] agree that three different levels of water stress can be considered: mild stress, when water shortages reach 8–10% of tissue water; moderate stress, when water shortage is 10–20% of tissue water; and severe or harsh stress, when water shortage is more than 20%. The negative effects can be even higher because of the intensity and duration of water stress [9]. Moreover, it is well known that water-resource availability fluctuates in space and time, which is also a problem within the soil–plant–atmosphere system, in which plant structure and function, as well as the whole ecosystem, can be affected by plant-to-plant competition [10]. This is a common issue in arid and semiarid areas that use irrigation by drop or flooding in order to mitigate the negative effects caused by weeds [11,12].

Since the mobilization and utilization of water resources by plants are governed by numerous interacting biotic and abiotic factors [13,14], investigations into field-crop production have focused on the search for management methods that can reduce the negative effects of water stress. These include improving crop varieties characterized by their resistance to stress; modifying irrigation regimes [15,16]; and using plant-growth regulators, fertilizers, and even plant extracts that enable the plant to conserve water or reduce transpiration, thereby increasing the osmotic potential of cells [17,18].

Some agricultural processes, such as tillage, mulching soil, and depth of cultivation, can increase water soil retention capacity to conserve water or increase the ability of the plant root to obtain the moisture required for plant processing [19,20]. One global strategy to reduce the impact of water deficits is to take advantage of sequence crop residues as cover on the soil surface, which is a practice aimed at improving the plant environment, conserving soil moisture, regulating soil temperature, minimizing salinization, and increasing the proportion of organic material, as well as suppressing weeds by reducing the pollution resulting from the use of chemical herbicides [21]. For instance, Hobbs et al. [22] noted that the use of cover crops is one of the most important agricultural practices in conserving soil moisture, cooling the surface, and modifying the physical environment, as it prevents the growth of weeds, mitigates drastic soil-temperature increases, and reduces the growth of roots of small plants outside the soil when it is freezing during winter. Crops such as wheat are also able to add organic matter to the soil if they are completely covered with organic natural materials, helping reduce erosion on slopes [23]. On the other hand, the use of crop residues, such as wheat, as mulch on the soil reduces the growth of annual weed seeds and the propagation of perennial species [24,25], which reduces the costs of control and the weed presence with the crop. Furthermore, the use of mulch and vegetation cover allows reducing soil and water losses caused by erosive events of tillage and extreme rain [26,27]. In this study, we used a wheat cover crop. In Iraq, wheat is considered an important plant species because of its low irrigation requirements, resistance against temperature changes, and availability in the region. In addition, there is a lack of information about the positive or negative effect of this species, which, as mentioned above, is usually also used to control weeds in other countries.

The use of postharvest residues of crops as a soil-surface cover in order to reduce the impact of drought and improve plant resistance to damage may have several benefits. These include positive results on the growth and production of medicinal crops, reduction of economic losses resulting from plant stress, and less need for irrigation in areas where water resources are scarce, particularly in undeveloped countries. However, the use of these residues could also lead to negative effects on the growth of the crop as a result of the release of allelochemical compounds to the soil [24]. Roth et al. [28] indicated that there is a significant effect of sorghum residues present as a cover crop on the growth and chlorophyll content of the next wheat crop, but that the effect is removed during the advanced crop stages due to the decomposition of these compounds and their transformation into organic matter. Therefore, information about the impact of cover crops on this topic is still unclear.

This study was designed to demonstrate the possibility of reducing the impact of water deficiency on the fenugreek crop through the use of wheat crop residues as a cover crop, the extent of its effects on chlorophyll during the growth stages, and the reflection in the yield of seeds and fixed oil for fenugreek.

## 2. Results

### 2.1. Weed Characterization

Estimating weeds in the field, the spread of broad-leaf species was noted. Of the total amount of weeds, 70% were composed of *Malva sylvestris* (20%), *Chenopodium album* (30%), *Sybmbrium septulatum* (10%), *Euphorbia tinctoria* (5%), and *Euphorbia helioscopia* (5%). Narrow-leaf weed species accounted for 30% of total plot t, with *Lolium temulentum* (15%), *Lolium rigidoum* (10%), and *Avena fatu* (5%).

**Figure 1:**
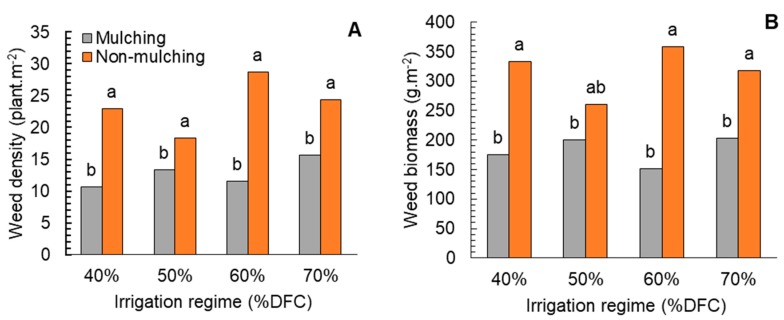
Effect of water depletion and mulching treatment on (**A**) weed density and (**B**) weed biomass at the physiological maturity stage of fenugreek. Means included within each bar followed by different letters showed significant differences at *p* < 0.05 DFC (%): Irrigation after depleting 40%, 50%, 60%, and 70% of field capacity.

Wheat-crop-residue mulching showed a significant reduction of weed density and dry biomass, while no significant effects of water stress on it were noted (Table 1). The surface-mulching wheat straw ensured a high suppression rate of weed density and dry biomass by 58.6% and 42%, respectively compared with the non-mulching treatment. Water-stress treatment did not show significant effects on weed growth and development, which showed a similar trend of weed density (16.8–20.15 plants per m^2^) and dry biomass (231.5–260.5 g·m^−2^). Interaction between mulching treatment and water stress showed a clear suppuration of weed density and biomass with all levels of depletion compared with the non-mulching treatment (Figure 1). 

### 2.2. Leaf Chlorophyll Content 

Combined analysis of variance related to the main effects of mulching and water-stress treatment indicated significant differences in chlorophyll content during the three different studied growth stages (Table 2). At 60 days after sowing (DAS), the chlorophyll of leaves decreased with mulching treatment by wheat straw (23.55 SPAD) compared to the non-mulching treatments (32.89 SPAD). This effect of mulching did not continue to 90 DAS, but was removed, and even improved leaf chlorophyll content, showing non-significant differences between mulching and non-mulching treatments (33.99 and 35.32 SPAD, respectively). At 140 DAS, specifically after the formation of pods (at the full seed stage), the impact was reflected between mulching and non-mulching treatment, as the treatment of soil mulching by wheat crop residues showed a higher chlorophyll content in the leaves (58.17 SPAD) compared to the control plot (38.85 SPAD).

Water stress (following irrigation-regime treatment) affected the content of chlorophyll at 60 DAS. Irrigation regime at 60% DFC had the highest values (35.63 SPAD), while the lowest chlorophyll content appeared under the highest water-stress conditions (70% DFC), amounting to 26.28 SPAD. At 140 DAS, the decline of chlorophyll was evident from high-moisture depletion (70% DFC). Leaf chlorophyll content did not show an impact at 90 DAS due to the water-regime treatment, but it was affected at 140 DAS, with the highest rates being 50% and 60% DFC (53.2 and 54.85 SPAD), respectively. Moreover, no significant differences with the normal treatment of irrigation (40% DFC) were obtained, showing a lower average of chlorophyll content (48.55 SPAD).

**Figure 2:**
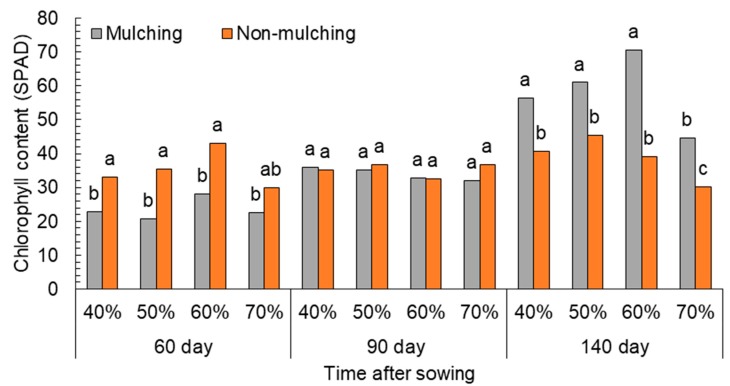
Effect of water depletion on leaf chlorophyll content. Means included within each bar followed by different letters showed significant differences at *p* < 0.05. DFC (%): Irrigation after depleting 40%, 50%, 60%, and 70% of field capacity.

Finally, the results of the interaction between mulching treatment and water-stress coefficients during the three growth stages (60, 90 and 140 DAS) are shown in Figure 2. Chlorophyll content was the highest in non-mulching treatments during the first 60 days. Then, at 90 DAS, the use of mulching did not differ in the results. However, at 140 DAS, the effect was reversed, with mulching treatment being higher than non-mulching for all water-stress levels.

**Figure 3:**
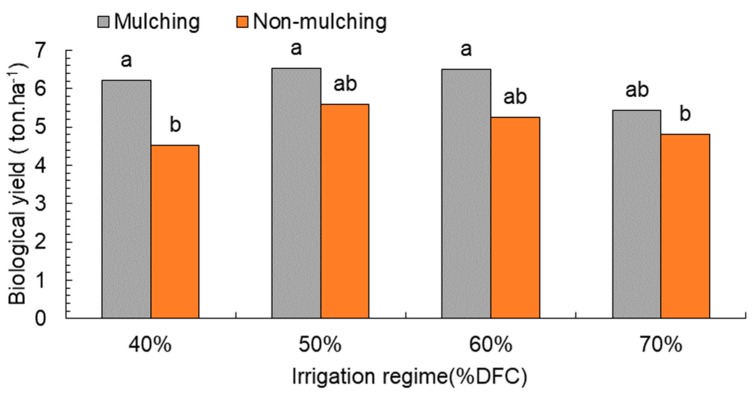
Effect of water depletion on biological yield (ton·ha^−1^). Means included within each bar followed by different letters showed significant differences at *p* < 0.05. DFC (%): Irrigation after depleting 40%, 50%, 60%, and 70% of field capacity.

Yield biology significantly increased when mulching treatment was used (6.17 ton·ha^−1^) compared to the non-mulching plot (5.04 ton·ha^−1^) (Table 3). The high-water-regime treatment (70% DFC) significantly decreased biological yield, while it increased with 50% and 60% DFC. The interaction between mulching and irrigation regime was similar among mulching-treatment regimes, while it was different from the non-mulching treatment (Figure 3).

Seed yield showed a significant increase from the mulching to non-mulching treatments. By using wheat straw as mulching crop, it was 1.25 ton·ha^−1^ compared to 0.86 ton·ha^−1^ by using the non-mulching treatment. Water-regime treatments also significantly affected seed yield. Therefore, moisture-depletion levels of 50% and 60% DFC obtained the highest yield of seeds per hectare (1.17 and 1.39 ton·ha^−1^). On the other hand, at 40% irrigation and high water stress (70% DFC), 0.89 and 0.76 ton·ha^−1^ were obtained, respectively (Table 4). 

**Figure 4:**
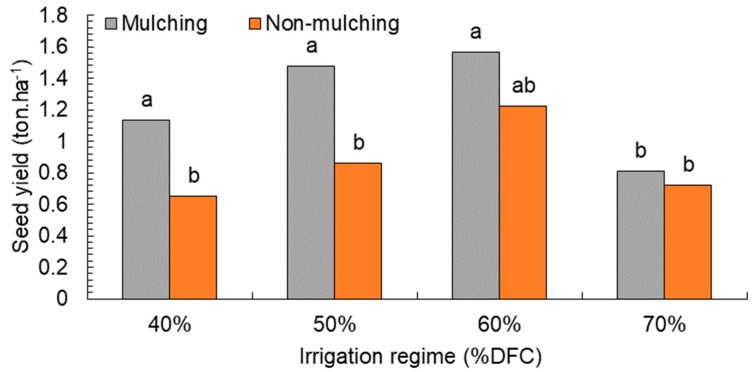
Effect of water depletion on seed yield (ton·ha^−1^). Means included within each bar followed by different letters showed significant differences at *p* < 0.05. DFC (%): Irrigation after depleting 40%, 50%, 60%, and 70% of field capacity.

According to Figure 4, seed yield was larger in the mulching treatment compared to the non-mulching plot with the lower levels of water stress (50% and 60% DFC), while there was a difference between mulching and non-mulching treatment when water depletion increased by more than 70%; however, we could observe that the produced seeds in mulching under 60% water stress surpassed irrigation at 40% DFC (control).

**Figure 5:**
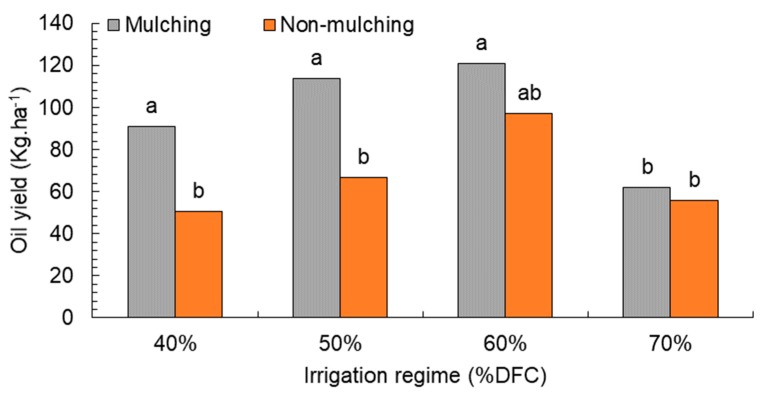
Effect of water depletion on oilseed yield (Kg·ha^−1^). Means included within each bar followed by different letters showed significant differences at *p* < 0.05. DFC (%): Irrigation after depleting 40%, 50%, 60%, and 70% of field capacity.

Finally, fixed seed oil was tested, and the results are shown in Table 3. In general, different levels of water deficit and mulching treatment significantly affected oil percentage. Mulching treatment led to 96.7 kg·ha^−1^, while for the control plot it was 67.5 kg·ha^−1^. Water-tress treatment influenced fenugreek oil production, with 50–60% DFC had the highest oil yield: 90.2 and 108.9 kg·ha^−1^, respectively. At 70% depletion, oil production of 58.9 kg·ha^−1^ was recorded. Treatments of water stress at 40% depletion produced 70.7 kg·ha^−1^ oil. The interaction between levels of water stress and mulching treatment (Figure 5) was also consistent with the increase of seed yield, as the increase was evident in the mulching compared to the non-mulching treatments at the three regime levels (40%, 50%, and 60% depletion). On the other hand, oil yield in both treatments decreased at 70% depletion.

## 3. Discussion

The leaf is the main part in which plant photosynthesis and metabolism are generated, so increasing the leaf area and chlorophyll content to the optimal levels helps to improve system constant capacity (SCC) [29], with positive consequences on quality and productivity. It is also well known that climate factors are key parameters for leaf growth; therefore, when soil-temperature and moisture conditions are agreeable, it also improves leaf growth [30]. Conversely, we observed in this study that water stress can decrease green-leaf growth and increase senescence, which may be reflected in decreased photosynthesis efficiency, as other authors have observed [16]. Therefore, introducing new environmentally friendly methods to conserve soil temperature and moisture is a key question.

Our results showed that the presence of wheat residues (straw) on the soil surface could negatively affect the growth of the fenugreek crop during the early stages due to the release of some inhibitory allopathic (allelochemical) compounds to the soil, thus reducing leaf chlorophyll content and decreasing photosynthesis efficiency. These compounds can be phytotoxins run of dead cells with water irrigation and absorbed by the roots of the crop [31,32]. Some researchers have pointed out that the majority of these substances are phenolic compounds that are water-soluble, and can access living tissue of plants and micro-organisms, inhibiting their vital efficacy [33,34]. This was also confirmed by Roth et al. [28] when they studied the impact of the soil-surface presence of sorghum residues on the chlorophyll content of the wheat plant. Khaliq et al. [35] also found that adding 4, 6, or 8. kg.m^−2^ of wheat residues on the soil surface decreased total leaf chlorophyll content by 40%, 45%, and 65% compared with control treatment. Although the current study did not address the measurement of these compounds, previous studies on the wheat crop in Iraq have diagnosed these compounds. Chemical analysis on these phenolics by high-performance liquid chromatography (HPLC) indicated the presence of several allelochemicals, viz, p-hydroxybenzoic, protocatechuic, vanillic, p-coumaric acid, ferulic acid, sinapic acid, and syringic acid [36].

As the growing season progressed to 90 days after sowing, there was no significant difference in leaf chlorophyll content between mulching and non-mulching treatments, which indicated the degradation of allelopathic compounds and removal from the soil, which was also demonstrated by some other researchers [37,38,39]. The effect of allelopathic compounds in the soil may last 8–10 weeks in soil, then leading to degradation by micro-organisms and transfer to organic matter. Albehadili et al. [40] also pointed out that allelopathic compounds, which are free of wheat crop residues, continue to have an effect on the soil after a few weeks, after which they can degrade and become useful organic materials for the harvest. In determining the total concentration of phenols in the field, it was observed on soil blended with wheat residues during different stages of decomposition that the concentration of these compounds was at its highest level at two weeks and a month from cultivation, before gradually decreasing six weeks after addition and fading at around eight weeks [38]. 

Our results also showed that weed germination and growth were clearly reduced during this period. This could confirm the apparent improvement in chlorophyll content with the presence of residues in the soil after the flowering phase and the formation of the cornea. This fact can confirm that allelopathic compounds and residues can be mineralized in the soil and increase the contents of ready-made nutrients, which would be reflected in an increase in the proportion of chlorophyll [41,42]. This result is consistent with Alsaadawi et al. [43], and Lahmod [32] who found the decomposition of plant residues to improve the growth of the wheat crop. Lahmod et al. [44] also confirmed that sunflower plant residues increased soil nutrients such as nitrogen, potassium, calcium, magnesium, sulfur, and organic matter, improving the growth of the broad-bean crop. Improvement in plant growth under the mulching system indicates the positive role that crop residues play in increasing yield. Stagnari et al. [44] noted that wheat-straw mulching results in enhanced moisture conservation, especially at a critical growth stage of the tested crop. Decomposed residues not only supplied allelochemicals but also participated in crop nutrition through nitrogen release in the plant rhizosphere. Immobilization of nitrogen is observed under biological mulch, which may decrease the immediate supply of nitrogen.

High-water-stress treatment (70% DFC) affected the first months of growth because severe water stress directly affects photodynamic systems and leads to the reduction of leaf-pigment content [16]. In late growth stages, the highest chlorophyll content was recorded with levels of water stress of 50% and 60% depletion, without significant difference from normal irrigation treatment (40% DFC). The reason for the low chlorophyll in 40% DFC may be due to the leaching of nutrients from the soil, especially nitrogen; as a result, the increase of irrigation during the season [45] is due to the nitrogen element being essential in the construction of chlorophyll molecules. On the contrary, the reduction of the irrigation period to a level that may not cause nutrient leaching and prevent chlorophyll degradation would inescapably enhance chlorophyll in leaves [20]. The high depletion of moisture in the soil (70%) would decrease chlorophyll and lead to chlorophyll decomposition as a result of the stress effect on the plant’s structural-process molecules [46].

Mulching treatments showed a clear influence on weed growth, while water stress did not affect the weeds. The presence of residues on the soil surface that could have caused a physical suppression of weed seedlings and prevent separated light from it [24,25] and the allelopathic role would affect residues in reducing weed growth [36]. The presence of crop residues on the soil surface can lead to the physical impairment of weed growth and the release of some allelopathic compounds from these residues with irrigation water, which were known effects on the growth of small weed seeds during the early stages of growth [47]. The release of these compounds could be related to some factors such as the type of cover crops, quantity or intensity of the cover, period of decomposition, soil moisture, and microflora [48,49]. This fact could confirm that the presence of allelochemical compounds contains the low chlorophyll content of the fenugreek crop during the early growth stages of the treatment of the presence of wheat residue. However, the low SPAD value in experiment combinations with mulch in the early stages of crop development can be due to many different reasons. Irrigation regime did not affect the growth of the weeds, which is probably the result of the ability of weeds to adapt to water-stress conditions more efficiently than crops can [50]. The low growth of the fenugreek crop due to drought gives a greater chance of competition and filling the place compared to 40% depletion, which helped them to be unaffected by high-stress conditions. The interaction between mulching treatment and irrigation-regime treatment recorded a weed density from 10.7 to 17.3 plant m², while this increased by twice for the non-mulching treatment. This result confirms the role of soil mulching in decreasing weeds, as confirmed by [22]. 

The effect of water stress and soil mulching was evident on the growth and yield of the fenugreek. The presence of wheat residues as soil-surface mulch helped to maintain soil moisture, which reduced the impact of water stress on the crop [30], and the low density of weeds due to the presence of the residue reduced water and nutrient competition in soil [25]. This positively reflects an increase in the biological yield of the crop.

Increased seed yield means the ability of plant species to convert as much of the output of photosynthesis into seeds, depending on the size of the sourced assimilates and the seed capacity to store them [18]. The results showed that the best yield of fenugreek seeds was recorded with the irrigation-regime treatment (50% and 60% DFC), which exceeded the normal irrigation treatment (at 40% DFC), while the higher depletion level affected seed yield (70% DFC). The decline in the treatment of normal irrigation (40% DFC) may indicate an increase in the amount of water irrigation from the plants need, thus increasing nutrient leaching and draining. The high-stress treatment (70% DFC) may be the result of the impact of stress on the activity and growth of cells and the production of plant dry matter [51,52,53].

In the current study, a more than 30% improvement in fenugreek yield was recorded through management by organic mulch. The improvement in seed yield might be attributed to weed suppression during critical crop-growth periods. Effective weed suppression enhanced the availability of growth factors such as nutrients, water, light, and space [54]. A study by Stagnari et al. [44] on wheat-straw mulching resulted in enhanced soil-moisture conservation at a critical growth stage of the wheat. Decomposed wheat straw not only supplied allelochemicals, but also participated in the crop-nutrition availability of nitrogen release in the plant rhizosphere. Immobilization of nutrients as nitrogen is observed in biological coverage crop, which may decrease the supply of nitrogen [54]. Moreover, the availability of nitrogen was enhanced at the later stage of plant growth through the mineralization process, so this prolonged supply is a continuous source for the plant during organic mulching treatment.

Fixed oil is an economic and important medical component of fenugreek, and may be associated with the number of seeds produced per unit area and the environmental factors influencing the life of the plant. Soil mulching of crop residues was recorded as an oil quotient in relation to non-mulching treatment due to the effect of mulching on increasing plant content from active substances through improved seed yield, and increased chlorophyll and soil-mineral-element content [24]. This, in turn, improves the process of photosynthesis and the production of carbohydrates, fats, proteins, and some secondary metabolic compounds of the active ingredient, thus increasing seed yield of the seed; meanwhile, the proportion of oil in the non-mulching treatment possibly decreased due to low photosynthesis level, increasing the evaporation or low absorption of mineral materials from the soil [55,56], and the spread of weed competition for the main growth elements.

Generally, moderate water deficit (50–60% DFC) was recorded as leading to a significant increase of seed and oil yield in the fenugreek crop under mulching conditions compared with the non-mulching treatment. This increase may be due to two basic factors: first, increases in soil moisture due to the reduction of evaporation processes; and second, decreases in weed density and competition. Increasing water stress to the 60% level under two treatment conditions (mulching and non-mulching) contributed to a gradual increase in oilseed yield. The reason for the increase in oil under conditions of moderate water stress may be due to the fact that a plant under water-stress conditions tends to increase the concentration of carbohydrate compounds and fats for the osmosis concentration of cells to resist droughts [57]. Alteration in secondary metabolite agents is also an important biomarker of crop defense against unfavorable conditions [58]. When it comes to medicinal plants, water deficit has been found to alter essential oil seed composition [59]. According to previous studies, secondary-metabolite synthesis in aloe vera increases under drought-stress conditions [57,60]; an increase in the secondary-metabolite component as a response to water deficit protects plants against adverse effects of water stress. Moreover, drought stress is a way of enhancing the plant contents of secondary products, thereby increasing the quality of plant-extract commodities [57].

## 4. Materials and Methods

### 4.1. Treatment and Experiment Design 

A field experiment was conducted in a split-plot design with 3 replicates in a field crop located at the Faculty of Agriculture of Wasit University in the southern region of Iraq (45.85E and 32.49N), during the winter season of 2016–2017. Four irrigation regimes, namely, after 40%, 50%, 60%, and 70% DFC, combined with 2 mulching wheat-straw treatments (mulching and non-mulching) were allocated to 3 randomized blocks (main plots). The main physical soil properties are outlined in Table 4.

### 4.2. Field Preparation

**Figure 6:**
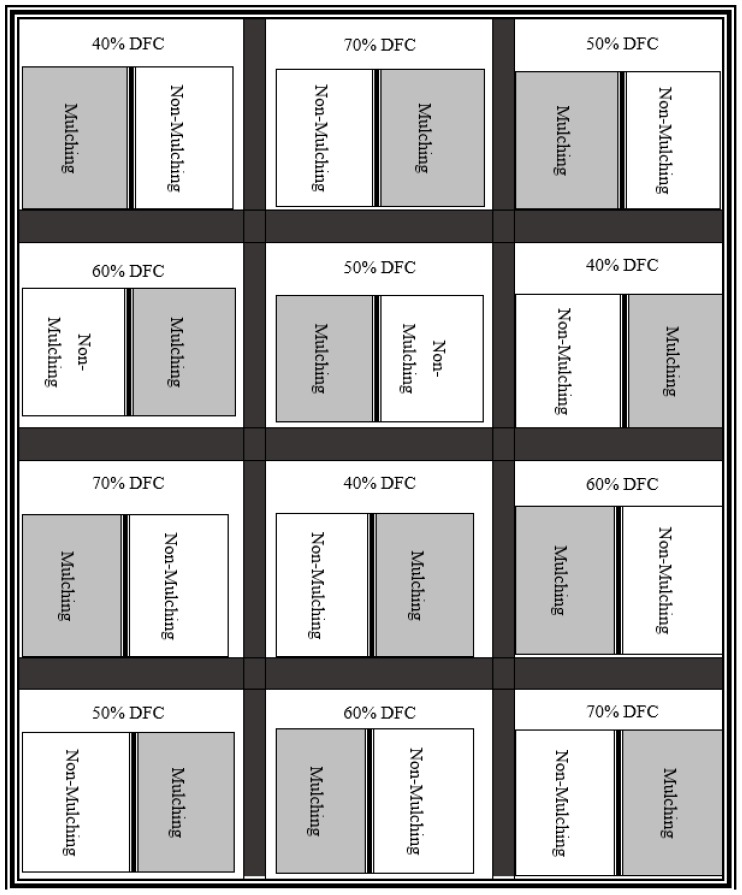
Flowchart of experiment design and treatment distribution. %DFC: Depletion from Field Capacity as a percentage.

**Figure 7:**
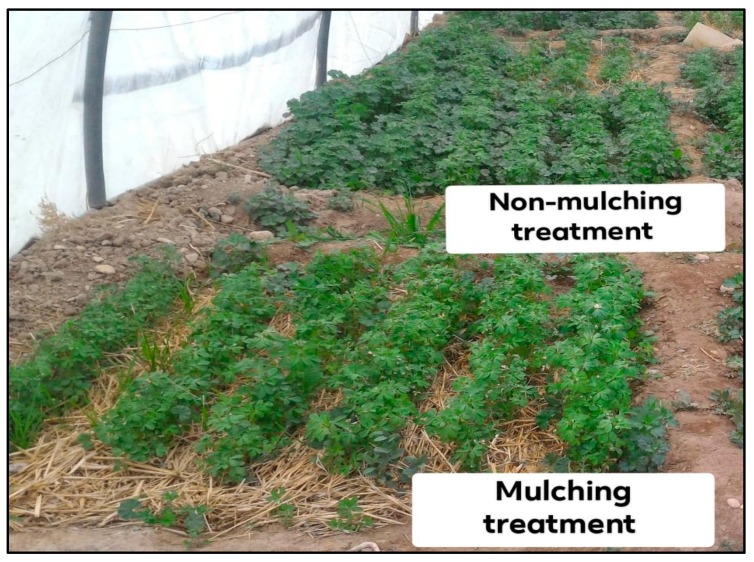
Mulching and non-mulching treatments.

The field was tilled and smoothed on 13 October 2017. Three replications for each plot represent the different irrigation regime types with 2 treatments of wheat straw as a cover crop (mulch) and without mulching (non-mulching) with a size of 2 × 2 m (Figure 6 and Figure 7). 

The fenugreek seeds were planted with distance lines of 25 cm at a rate of 160 kg per ha. The cultivation and addition of 200 kg·ha^−1^ of Nitrogen, Phosphorus and Potassium (NPK) (18-18-0) were done on 10 October 2017, while urea fertilizer was added at a rate of 90 kg per ha [61], which was divided into 2 stages: adding 2 weeks after planting, and replication again after 2 months per experiment unit.

### 4.3. Irrigation-Regime Treatment

The experiment included 4 irrigation treatments: usual irrigation treatment (control), after depletion of 40% DFC, and 3 levels of water stress (50%, 60%, and 70% DFC). Irrigation was done via a flexible plastic piping system with a 2-inch diameter attached to a water pump that was calibrated with a water-flow meter and calculated depending on the depletion of water content in each control treatment. The irrigation process was carried out on the basis of depth depletion, which was from 0 to 20 cm until the vegetative stage. Then, the depth of irrigation water was increased to 30 cm of soil depth at the beginning of the flowering stage, and then to 40 cm soil depth at the end of physiological maturity to enhance access to field capacity. Field capacity and available water were estimated using soil-texture hydraulic-property calculator software according to [62]. To determine irrigation dates and quantities, moisture was measured by the weight method of soil depths before each instance of irrigation to determine the date of irrigation for each treatment. Controlling the amount and quality of water used for this experiment was considered as a priority because of the serious-related droughts and pollutants close to the urban areas in this arid territory [63,64].

Available water was determined through the range between moisture at field capacity and the wilting point of the soil. Determination of water depth to be added to compensate for field-capacity depletion was calculated by using the following equation [65]:

d = [θf.c − θbi ] D,(1)

where d is the depth of water addition (cm), θF.c represent the moisture at field capacity, θbi is the volumetric moisture before irrigation, and D is plant-root depth (cm). Then, the water depth of the next irrigation was determined according to Equations (2) and (3):

D = D × ΔӨ(2)

D × (Өfc − Өd),(3)

where Өd is Өfc – depletion rate x available water, d represents the depth of irrigation water (cm), and Өd the moisture content that caused the depletion. The volume of water adding to each plot was calculated according to Equation (4):

V = d × A,(4)

where V is the volume of added water and A is the plot area (m^2^).

### 4.4. Weed Characterization

#### 4.4.1. Weed Density (Plant m^−2^)

A 1 m^2^ microplot was randomly placed on plots at the physiological-maturity stage of fenugreek to record weed density. The average was calculated and converted to weed density per squared meters. 

#### 4.4.2. Weed Biomass (g·m^−2^)

The weeds were counted at the physiological maturity stage of fenugreek, cut from the ground surface, and then brought to the laboratory to weigh and record their biomass. Their total dry weight was determined after oven drying at 75 °C for 3 days until a constant weight was achieved. The average was calculated on a square-meter basis.

### 4.5. Leaf Chlorophyll Content and Yield Characterization 

Leaf chlorophyll content was estimated by the chlorophyll-measuring device 502 SPAD, processed by the Japanese company Minolta, by reading 10 sheets of the plate, and then calculating the average measurements by SPAD (nano mol per cm^2^) [66]. Chlorophyll content was measured for 3 stages of crop growth 60, 90, and 140 days after sowing. 

Biological yield (tons per ha) was calculated by drying the crop biomass per m^2^ after harvesting and then weighing. Then, seed yield (tons per ha) was estimated after harvesting the fenugreek plant per m^2^ and then multiplying by 10,000 as yield per hectare. Fixed oil yield (kg per ha) was obtained by using a sixolite device, diluted ethanol (75/25 mL alcohol/water), and 1 g of seed powder.

### 4.6. Statistical Analysis

The data were statistically analyzed with analysis of variance (ANOVA) with the GEN STAT software package. Differences between treatments were compared through the Least Significant Difference (LSD) test at the ≤0.05 probability level.

## 5. Conclusions

Through the results, it could be inferred that the use of wheat residues as a cover crop on the soil surface can improve soil’s conservation moisture. Moreover, it could reduce weed growth and improve the growth and productivity of the fenugreek crop, which is essential for oil production. Although there was a negative impact of allelochemical compounds freed from these residues in the early growth stages, this effect could quickly be removed during the advanced stages of crop growth. Therefore, we recommend the use of wheat straw as a cover crop, but only if the field is irrigated at least two weeks before because of the negative effect of allelochemicals or the activity of micro-organisms through adding nitrogen fertilizer.

## Figures and Tables

**Table 1:** Main effects of irrigation regime and mulching treatment on weed and yield production.

	Weed Density	Weed Biomass
	(plant·m^−2^)	(g·m^−2^)
Mulching	13.82 b	182.5 b
Non-mulching	23.57 a	317.5 a
Irrigation regime (after depleting %FC)		
40% DFC	16.85 a	254.0 a
50% DFC	17.8 a	231.0 a
60% DFC	20.15 a	254.5 a
70% DFC	20.00 a	260.5 a
C.V	20.5	19.3
Means within a column followed by the same letter are not significantly different (*p*-value ≤ 0.05).		

**Table 2:** Main effects of irrigation regime and mulching treatment on leaf chlorophyll content.

	Chlorophyll Content of Leaves (SPAD)		
	Time after Sowing (Day)		
	60 days	90 days	140 days
Mulching	23.55 b	33.99 a	58.17 a
Non-mulching	32.89 a	35.32 a	38.85 b
Irrigation regime (after depleting %FC)			
40% DFC	27.98 b	35.62 a	48.55 a
50% DFC	28.00 b	35.88 a	53.20 a
60% DFC	35.63 a	32.61 a	54.85 a
70% DFC	26.28 b	34.50 a	37.45 b
C.V	13.3	8.8	14.8
Means within a column followed by the same letter were not significantly different (*p* ≤ 0.05).			

**Table 3:** Main effects of irrigation regime and mulching treatment on fenugreek weed and yield.

	Biological Yield	Seed Yield	Oil Yield
	(ton·ha^−1^)	(ton·ha^−1^)	(Kg·ha^−1^)
Mulching	6.17 a	1.25 a	96.7 a
Non-mulching	5.04 b	0.86 b	67.5 b
Irrigation regime (after depleting %FC)			
40% DFC	5.36 b	0.89 b	70.7 bc
50% DFC	6.06 a	1.17 a	90.2 ab
60% DFC	5.88 ab	1.39 a	108.9 a
70% DFC	5.12 b	0.76 b	58.9 c
C.V	15.8	18.5	18.7
Means within a column followed by the same letter were not significantly different (p ≤ 0.05).			

**Table 4:** Plot physical properties.

Sand	Silt	Clay	Wilting Point	Field Capacity	Available Water	Saturation	Bulk Density	Hydraulic Conductivity
%			by volume			%	mg/m^3^	cm/h
36	47	17	0.11	0.26	0.15	0.46	1.4	1.2
